# New insights into the gut-heart axis: microbial metabolite biomarkers, multi-omics, and engineered bacterial delivery systems

**DOI:** 10.3389/fmicb.2026.1987882

**Published:** 2026-09-28

**Authors:** Yankun Chen, Yiyu Li, Huaguan Lu, Shulei Jia, Yajie Jiang

**Affiliations:** 1 Engineering Research Center of Innovative Drug of Traditional Chinese and Zhuang Yao Medicine, Ministry of Education, Guangxi University of Chinese Medicine, Nanning, China; 2 Department of Medical Biochemistry and Molecular Biology, School of Basic Medicine, Guangxi Medical University, Nanning, China; 3 Key Laboratory of Biological Molecular Medicine Research (Guangxi Medical University), Education Department of Guangxi Zhuang Autonomous Region, Nanning, China; 4 Institute of Grand Health, Guangxi Academy of Sciences, Nanning, China; 5 School of Basic Medical Sciences, Tianjin Medical University, Tianjin, China

**Keywords:** biomarkers, cardiovascular disease, gut microbiota, gut-heart axis, multi-omics

## Abstract

Despite rapid growth in gut–heart axis research, clinical translation remains limited. This review addresses that gap by critically synthesizing recent evidence on microbiota-derived metabolites, biomarkers, multi-omics workflows, and engineered bacterial interventions in cardiovascular disease (CVD). We examine how TMAO, short-chain fatty acids, bile acids, indole derivatives, and phenylacetylglutamine exert context-dependent cardiovascular effects, and why functional microbial gene markers and integrated biomarker panels may outperform single-taxon signatures. We then evaluate multi-omics strategies, metagenomics, metabolomics, proteomics, single-cell and spatial omics, and bioinformatics integration, for causal inference and biomarker validation. Finally, we assess engineered bacterial delivery systems, including gene circuit design, colonization control, biocontainment, and safety, and outline short-, medium-, and long-term milestones for clinical translation, cost-effectiveness, and intellectual property. The key takeaway is that precision microbiome medicine in CVD will require standardized assays, prospective validation, robust regulatory pathways, and equitable commercialization. By linking mechanistic insights with translational requirements, this review provides a framework for developing microbiome-targeted diagnostics and therapeutics in CVD.

## 1 Introduction

Cardiovascular diseases (CVDs) remain the leading cause of mortality and morbidity worldwide. Despite advances in diagnosis and treatment, residual cardiovascular risk persists after optimal control of traditional risk factors. The gut microbiota has emerged as a key contributor to host metabolism, immune regulation, and vascular function, forming the concept of the gut–heart axis (Sethi et al., 2023; Kumar et al., 2022). Alterations in microbial composition and function, termed dysbiosis, have been implicated in atherosclerosis, heart failure, and arrhythmias (Fan et al., 2023; Anwar et al., 2022). Although bacteria are the most studied component, the gut virome and mycobiome also influence host immunity and cardiovascular risk (Shkoporov and Hill, 2019; Iliev and Leonardi, 2017). This review focuses on the mechanistic roles of microbial metabolites, biomarker development, multi-omics integration, and engineered bacterial therapeutics, and outlines translational milestones for precision microbiome medicine in CVD. The detailed mechanistic pathways of TMAO, SCFAs, and bile acids, including their biosynthesis, receptor signaling, and cardiovascular effects, are systematically discussed in section 2 (Li et al., 2023; Yadav et al., 2022; Bilson et al., 2024).

Despite these advances, research on the gut-heart axis faces significant challenges. The gut microbiota exhibits substantial interindividual variability influenced by genetics, diet, environment, and comorbidities, complicating the identification of universal microbial signatures associated with cardiovascular risk. Furthermore, standardization of metabolite detection methods and validation of causal relationships remain critical hurdles. The complexity of microbial-host interactions necessitates integrative multi-omics strategies combining metagenomics, metabolomics, transcriptomics, and proteomics to unravel the mechanistic underpinnings and identify robust biomarkers (Sethi et al., 2023). High-throughput functional validation platforms are also essential to translate these findings into clinical applications.

In this context, synthetic biology and engineered bacterial delivery systems represent innovative tools to modulate the gut microbiome and its metabolic outputs with precision. Engineered probiotics can be designed to produce therapeutic molecules *in situ* or to modulate specific metabolic pathways implicated in cardiovascular diseases, offering a promising avenue for targeted intervention within the gut-heart axis (Fan et al., 2023). The rapid development of these technologies holds potential for personalized microbiome-based therapies that complement existing cardiovascular treatments. Although optimal control of traditional risk factors, a substantial residual cardiovascular risk remains, and current therapies do not adequately target microbiota-driven mechanisms. This unmet clinical need motivates the present review. We aim to critically synthesize the gut-heart axis from four perspectives: (i) microbial metabolites as mediators and biomarkers; (ii) multi-omics integration for mechanism and biomarker discovery; (iii) engineered bacterial delivery systems for precise modulation; and (iv) clinical translation, cost-effectiveness, and commercialization. We do not attempt an exhaustive catalog of all microbiota-CVD associations; instead, we focus on actionable pathways and translational barriers.

## 2 Evolution of the gut–heart axis concept

The concept of the gut–heart axis has evolved from early proof-of-concept studies linking gut microbial metabolites to cardiovascular risk into a comprehensive mechanistic framework. This section first describes the anatomical and functional connections between the gut microbiota and the host cardiovascular system, then discusses association and causality, and finally outlines causal inference frameworks and evidence grading.

### 2.1 Anatomical and functional connections between gut microbiota and host cardiovascular system

The gut-heart axis represents a complex and dynamic interplay between the intestinal microbiota and the host cardiovascular system, mediated through multiple anatomical and functional pathways. Central to this interaction is the gut mucosal barrier, which interfaces directly with the vascular system via the portal vein and mesenteric lymphatic circulation. This anatomical arrangement establishes a direct conduit for microbial metabolites and components to enter systemic circulation and reach distant cardiovascular tissues. The portal vein transports absorbed microbial metabolites such as SCFAs, bile acids, and extracellular vesicles (EVs) derived from gut bacteria, enabling these molecules to exert systemic effects on cardiac and vascular cells. For instance, recent studies have demonstrated that bacterial EVs from *Lachnospiraceae* can improve cardiac function post-myocardial infarction by enhancing intestinal stem cell function and maintaining gut barrier integrity, thereby reducing systemic inflammation and adverse cardiac remodeling (Chen et al., 2026). The mesenteric lymphatic system similarly facilitates the trafficking of immune cells and microbial antigens, contributing to immune surveillance and systemic immune modulation.

In parallel, the gut-associated lymphoid tissue (GALT) plays a pivotal role in maintaining systemic immune homeostasis through continuous low-level stimulation by microbial antigens. This persistent antigenic exposure shapes the host immune response, promoting tolerance under physiological conditions. However, dysbiosis or disruption of the gut barrier can lead to aberrant translocation of microbial products such as lipopolysaccharide (LPS), triggering systemic inflammation that contributes to endothelial dysfunction and destabilization of atherosclerotic plaques. This mechanism underscores the immunological axis of gut-cardiovascular communication, where microbial dysbiosis-induced immune activation exacerbates vascular injury and promotes cardiovascular disease progression. Beyond the portal vein and mesenteric lymphatics, the enteric nervous system (ENS) provides a dense neural network that bidirectionally communicates with the central nervous system and can modulate cardiac autonomic tone, vascular reactivity, and inflammatory reflexes. Microbial metabolites and endotoxins can influence ENS activity, thereby indirectly affecting cardiovascular function.

Moreover, the gut-heart axis extends beyond humoral and immune pathways to include neurogenic communication via the enteric nervous system and the vagus nerve. These autonomic neural circuits establish bidirectional signaling between the gut microbiota and central cardiovascular regulatory centers in the brainstem, forming a microbiota-gut-brain-heart network. This neuroimmune crosstalk modulates heart rate, vascular tone, and inflammatory responses, broadening the traditional concept of the gut-heart axis from a unidirectional metabolic pathway to a complex integrative system. Collectively, these anatomical and functional connections highlight the multifaceted mechanisms by which gut microbiota influence cardiovascular physiology and pathology, providing a foundation for therapeutic strategies targeting the microbiota-gut-heart axis (Chen et al., 2026; Zhang et al., 2024; Hou et al., 2026). Gut barrier integrity is increasingly implicated in CVD. Zonulin, a regulator of tight junctions, is elevated in conditions associated with increased intestinal permeability. When the barrier is compromised, LPS and bacterial components translocate into the circulation, triggering low-grade systemic inflammation, endothelial dysfunction, and adverse cardiac remodeling.

### 2.2 Association, causality, and causal inference in the gut–heart axis

Accumulating evidence from metagenomic and functional studies has established a strong association between gut microbiota composition and cardiovascular disease risk. Large-scale metagenome-wide association studies (MWAS) have consistently identified reduced microbial diversity and depletion of beneficial commensal genera such as *Roseburia* and *Faecalibacterium* in patients with coronary artery disease, heart failure, and hypertension. Large population-based cohorts, including the Zhernakova cohort and other metagenomic studies in atherosclerotic cardiovascular disease, have provided robust evidence linking gut microbial composition and functional potential to cardiovascular risk factors and disease phenotypes (Zhernakova et al., 2016; Jie et al., 2017; Karlsson et al., 2012). These taxa are known producers of anti-inflammatory SCFAs, and their diminished abundance correlates with heightened systemic inflammation and endothelial dysfunction, key drivers of CVD pathogenesis. Such reproducible microbial signatures across diverse cohorts underscore the robustness of microbiota alterations as biomarkers for cardiovascular risk stratification.

Beyond observational correlations, experimental models provide compelling causal evidence linking gut microbiota to cardiovascular outcomes. Germ-free mice and antibiotic-treated animals exhibit altered blood pressure regulation and impaired myocardial repair, demonstrating the necessity of microbiota for normal cardiovascular homeostasis. These findings establish a direct mechanistic role of gut microbiota in modulating cardiovascular function and disease progression.

### 2.3 Causal inference frameworks and evidence grading

Establishing causal relationships between gut microbiota, microbial metabolites, and cardiovascular disease nevertheless requires triangulation across complementary study designs, because no single approach is sufficient. Mendelian randomization (MR) uses genetic variants associated with microbial taxa or metabolites as instrumental variables to reduce confounding and reverse causation (Davey Smith and Hemani, 2014; Burgess et al., 2019). For example, bidirectional MR has been used to assess the causal direction between TMAO and cardiovascular events, although findings remain mixed and depend on instrument strength and pleiotropy (Jia et al., 2019). MR studies have also explored causal links between gut microbiota, short-chain fatty acids, and metabolic traits (Sanna et al., 2019). FMT provides experimental evidence of transmissibility; transplantation of dysbiotic microbiota from hypertensive or atherosclerotic donors into germ-free or antibiotic-treated recipients can induce cardiovascular phenotypes, supporting a causal role of the microbiome (Gregory et al., 2015). However, FMT studies are limited by donor variability, small sample sizes, species differences, and safety concerns. Human challenge studies, such as dietary choline or carnitine challenge, can demonstrate acute metabolite responses and provide mechanistic insight into TMAO production (Tang et al., 2013). However, they typically assess short-term metabolic endpoints rather than hard cardiovascular outcomes, and ethical and practical constraints limit their long-term application. Confidence in causality increases when MR, FMT, longitudinal cohorts, and mechanistic animal models converge. Conversely, inconsistency across these approaches should prompt caution rather than premature causal claims (Table 1).

**Table 1:** Strength of evidence for major microbial metabolites in cardiovascular disease.

Metabolite	Source	Main cardiovascular effect	Human evidence	Animal evidence	MR support	Therapeutic implication
TMAO	Choline, carnitine, betaine	Pro-atherogenic, pro-thrombotic	Strong	Strong	Mixed/limited	TMA lyase inhibitors, diet
SCFAs	Fiber fermentation	Anti-inflammatory, vasoprotective	Moderate–strong	Strong	Limited	Fiber, probiotics, engineered bacteria
Bile acids	Microbial modification of primary bile acids	FXR/TGR5 signaling, metabolic regulation	Moderate	Strong	Limited	FXR/TGR5 modulators
Tryptophan derivatives	Dietary tryptophan	AhR-mediated immune/vascular effects	Emerging	Moderate	Limited	AhR-targeted approaches
PAGln	Phenylalanine	Platelet activation, heart failure	Moderate	Moderate	Emerging	Adrenergic/platelet modulation

The time required for gut microbiota changes to cause cardiovascular damage is highly variable: acute shifts in microbial metabolites, such as TMAO, LPS, and proinflammatory cytokines, can occur within days to weeks and may trigger endothelial dysfunction, platelet hyperreactivity, and thrombotic events, whereas structural vascular damage, including atherosclerosis and adverse cardiac remodeling, usually develops over months to years of chronic exposure. The latency also depends on diet, host genetics, baseline inflammation, and comorbidities; therefore, both acute and chronic timescales should be considered when designing microbiome-targeted interventions (Davey Smith and Hemani, 2014; Burgess et al., 2019; Jia et al., 2019; Sanna et al., 2019; Gregory et al., 2015; Tang et al., 2013; Vujkovic-Cvijin et al., 2020; Falony et al., 2016).

However, the functional redundancy inherent in complex microbial communities challenges the interpretation of single-species analyses. Many microbial taxa share overlapping metabolic capabilities, suggesting that focusing solely on taxonomic shifts may overlook critical functional pathways. Therefore, integrating metagenomic data with metabolomic and transcriptomic profiling to elucidate microbial gene functions and metabolic networks offers a more reliable approach to deciphering causal mechanisms. For example, targeting microbial metabolic pathways involved in bile acid transformation or SCFA production may reveal actionable nodes for intervention. This systems biology perspective is essential for constructing a comprehensive causal chain linking gut microbiota alterations to cardiovascular pathology and for developing precision microbiome-based therapeutics (Chen et al., 2026; Zhang et al., 2024; Hou et al., 2026).

Current causal evidence remains limited by several factors. First, observational associations are vulnerable to confounding by diet, age, sex, medication use, comorbidities, and socioeconomic factors (Vujkovic-Cvijin et al., 2020; Falony et al., 2016). Second, reverse causation is difficult to exclude: cardiovascular disease and its treatments can alter gut microbiota composition and metabolite profiles. Third, MR estimates may be biased by weak instruments, horizontal pleiotropy, population stratification, and winner’s curse (Davey Smith and Hemani, 2014; Burgess et al., 2019). Fourth, FMT studies in CVD are few, heterogeneous, and often underpowered; donor microbiota engraftment varies widely. Fifth, human challenge studies focus on acute metabolite changes and cannot establish long-term cardiovascular benefit or harm. Sixth, animal models exhibit species-specific differences in bile acid receptors, SCFA receptors, and immune-metabolic responses, limiting direct translation to humans (Table 1).

Finally, large-scale randomized controlled trials with hard cardiovascular endpoints are still lacking for most microbiome-targeted interventions. Therefore, current evidence should be interpreted as supportive but not definitive. Future studies should integrate MR, FMT, human challenge, longitudinal multi-omics, and adequately powered RCTs to establish causality with greater confidence (Table 1).

Having established the anatomical, immunological, and causal frameworks linking gut microbiota to cardiovascular physiology, we now turn to the principal molecular mediators of this axis. Section 2 examines the dual roles of microbial metabolites, both detrimental and protective, in cardiovascular health and disease.

## 3 The dual role of microbial metabolites in cardiovascular physiology and pathology

### 3.1 TMAO as a clinical risk biomarker derived from dietary precursors

Trimethylamine-N-oxide is a pivotal microbial metabolite linking diet, gut microbiota, and cardiovascular disease risk. TMAO is generated through a two-step process beginning with dietary intake of choline, L-carnitine, and betaine, nutrients abundant in red meat, eggs, and dairy products. These substrates are metabolized by gut microbial enzymes, particularly TMA lyases, to produce TMA, which is subsequently absorbed and oxidized in the liver by flavin-containing monooxygenase 3 (FMO3) to form TMAO (Al Samarraie et al., 2023; Zhen et al., 2023). This metabolic pathway underscores the critical role of gut microbiota composition and activity in modulating systemic TMAO levels (Table 2; Figure 1). Inter-individual variability in FMO3 activity, driven by genetic polymorphisms, sex, and hormonal status, substantially influences circulating TMAO levels.

**Table 2:** Major microbial metabolites in cardiovascular disease: source, function, evidence level, and therapeutic implications.

Metabolite	Source/precursor	Key microbial/Host factors	Cardiovascular mechanism/effect	Evidence level	Therapeutic implications	References
TMAO	Dietary choline, L-carnitine, betaine; microbial TMA; hepatic FMO3	CutC/D TMA lyases; FMO3	Pro-atherogenic and pro-thrombotic; activates MAPK/NF-κB, VCAM-1/ICAM-1, monocyte recruitment, foam cells; increases platelet Ca^2+^; exacerbates myocardial fibrosis	Large prospective cohorts/meta-analyses; causality confounded by diet and renal function	TMA lyase inhibitors, reduced TMA-precursor diet, fiber-rich diet, probiotics; large RCTs still lacking	Al Samarraie et al. (2023), Zhen et al. (2023), Adiga et al. (2026), Yu et al. (2026), Sanchez-Gimenez et al. (2022), and Chang et al. (2021)
SCFAs	Microbial fermentation of dietary fiber	GPR41/GPR43; HDAC inhibition	Anti-inflammatory, vasodilatory, metabolic regulation; reduced renin, improved endothelial-dependent vasodilation, tight-junction upregulation, reduced LPS translocation	Clinical intervention and association	Fiber/prebiotics/probiotics; SCFA-producing engineered bacteria; butyrate supplementation	Al Samarraie et al. (2023), Adiga et al. (2026), Yu et al. (2026), Shi et al. (2026), Morillas-Armenta et al. (2025), Chulenbayeva et al. (2025), Iorra et al. (2026), Un and Buys (2015), O'Riordan et al. (2022), and Kulkarni and Gaikwad (2025)
Bile acids	Liver synthesis; microbial BSH; 7α-dehydroxylation	FXR; TGR5; BSH; 7α-dehydroxylase	FXR regulates cholesterol, triglyceride, and glucose; TGR5 induces NO and suppresses inflammation	Mechanistic and preclinical; clinical association	Probiotic modulation of BSH; FXR/TGR5 agonists or antagonists	Bilson et al. (2024), Al Samarraie et al. (2023), Adiga et al. (2026), and Yu et al. (2026)
Tryptophan/indole derivatives	Dietary tryptophan	AhR; microbial indole enzymes	Biphasic AhR effects; barrier integrity, immune homeostasis, vascular tone; chronic activation linked to calcification and dysfunction	Preclinical/mechanistic	Dietary tryptophan modulation; probiotic strains; tissue-selective AhR modulators	Adiga et al. (2026) and Yu et al. (2026)
PAGln	Phenylalanine; microbial phenylacetic acid; host glutamine conjugation	ADRA2A/ADRB2	Enhances platelet integrin αIIbβ3 activation, granule secretion, thrombosis; heart failure severity	Clinical association; emerging mechanistic	Adrenergic antagonists; microbial phenylalanine metabolism inhibitors	Yu et al. (2026) Song et al. (2024), and Romano et al. (2023)
TMA	Choline, L-carnitine, betaine; CutC/D	TMA lyases	Immediate TMAO precursor; possible independent effects	Mechanistic	TMA lyase inhibitors	Zhen et al. (2023) and Chang et al. (2021)
Zonulin/LPS	Gut barrier dysfunction	Tight junctions; LPS translocation	Endothelial dysfunction, inflammation, plaque destabilization	Clinical association	Barrier-protective strategies	Chen et al. (2026) and Shi et al. (2026)

**Figure 1:**
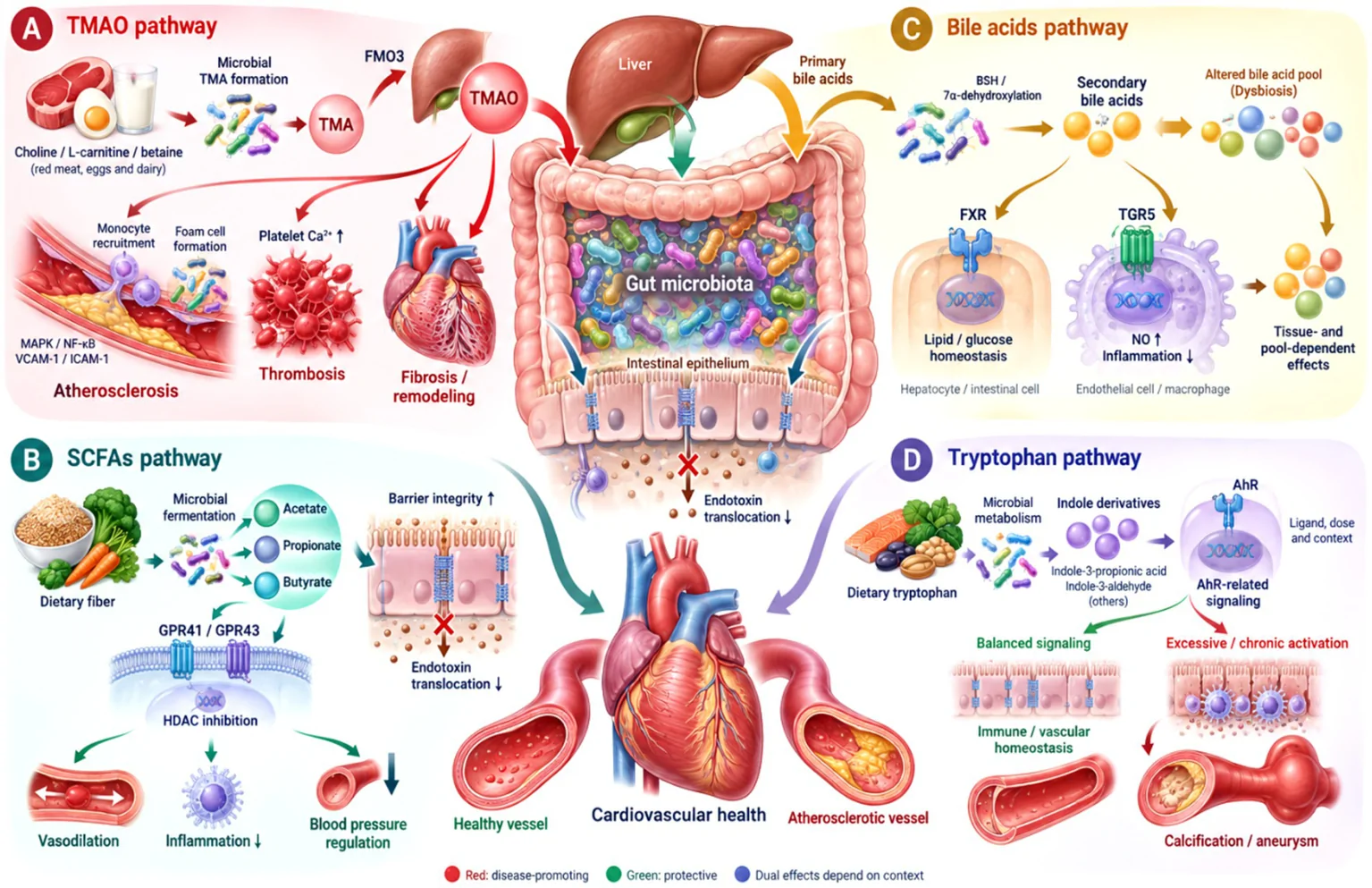
The major microbial metabolite pathways and their context-dependent cardiovascular effects. This figure illustrates how dietary precursors are converted by gut microbiota and host enzymes into TMAO, SCFAs, bile acids, and indole derivatives, and how these metabolites exert context-dependent protective or pathogenic effects on the heart and vasculature.

Mechanistically, TMAO exerts pro-atherogenic and pro-thrombotic effects through multiple molecular pathways. It activates mitogen-activated protein kinase (MAPK) and nuclear factor-kappa B (NF-κB) signaling cascades in vascular endothelial cells, leading to upregulation of adhesion molecules such as VCAM-1 and ICAM-1. This promotes monocyte recruitment and foam cell formation, key events in atherogenesis (Adiga et al., 2026; Yu et al., 2026). Additionally, TMAO enhances platelet reactivity by increasing intracellular calcium release in response to agonists, thereby elevating thrombosis risk (Zhen et al., 2023; Yu et al., 2026). In animal models of heart failure, elevated TMAO exacerbates myocardial fibrosis and adverse cardiac remodeling, implicating it in disease progression beyond atherosclerosis (Orjichukwu et al., 2025; Hatamnejad et al., 2025).

Large prospective cohorts have consistently associated circulating TMAO with major adverse cardiovascular events and all-cause mortality, supporting its role as a risk biomarker (Zhen et al., 2023; Sanchez-Gimenez et al., 2022). Recent professional society consensus documents have further supported the prognostic relevance of TMAO and related gut-derived metabolites in cardiovascular risk assessment (Yang et al., 2025). However, causal inference remains challenging due to confounding dietary and metabolic factors.

Therapeutically, targeting the TMAO biosynthetic pathway offers promising avenues. Preclinical studies demonstrate that inhibition of microbial TMA lyase enzymes (CutC/D) using small molecule inhibitors such as 3,3-dimethyl-1-butanol (DMB) effectively reduces TMA and TMAO production without disrupting overall microbiota balance or inducing resistance (Zhen et al., 2023; Chang et al., 2021). Dietary interventions, including reduced intake of TMA precursors and increased consumption of fiber-rich plant-based foods, modulate gut microbial composition and lower TMAO levels (Feng et al., 2026; Shi et al., 2026). Probiotic supplementation targeting TMA-producing bacteria has also shown potential in preclinical models. Nevertheless, translation into clinical practice is limited by a paucity of large-scale randomized controlled trials confirming efficacy and safety in humans (Adiga et al., 2026; Yu et al., 2026). Overall, TMAO exemplifies a gut microbiota-derived metabolite with a dual role as a mechanistic contributor to cardiovascular pathology and a clinically relevant biomarker, highlighting the gut-heart axis as a frontier for novel diagnostic and therapeutic strategies.

### 3.2 Protective regulatory network of short-chain fatty acids

The Short-chain fatty acids, primarily acetate, propionate, and butyrate, are key beneficial metabolites produced by the fermentation of dietary fibers by gut microbiota. These SCFAs constitute a protective metabolic network within the gut-heart axis, exerting anti-inflammatory, vasodilatory, and metabolic regulatory effects that counterbalance pro-atherogenic processes (Al Samarraie et al., 2023; Adiga et al., 2026; Table 2; Figure 1). Their biological actions are mainly mediated through activation of G protein-coupled receptors GPR41 and GPR43, as well as inhibition of histone deacetylases (HDACs), which modulate gene expression epigenetically (Morillas-Armenta et al., 2025; Chulenbayeva et al., 2025). GPR41 (FFAR3) and GPR43 (FFAR2) exhibit distinct ligand preferences and tissue distributions. GPR41 is more responsive to propionate and butyrate and is highly expressed in adipose tissue and enteric neurons, whereas GPR43 is activated by acetate and propionate and is enriched in immune cells and intestinal epithelium.

Activation of GPR41/43 by SCFAs leads to decreased renin secretion, improved endothelial-dependent vasodilation, and reduced release of pro-inflammatory adipokines, collectively contributing to blood pressure regulation and attenuation of atherosclerosis (Adiga et al., 2026; Yu et al., 2026). Butyrate, in particular, serves as the primary energy source for colonocytes and enhances intestinal barrier integrity by upregulating tight junction proteins, thereby reducing systemic translocation of endotoxins such as lipopolysaccharide (Yu et al., 2026; Shi et al., 2026) This barrier maintenance indirectly mitigates chronic low-grade inflammation implicated in cardiovascular pathology (Table 2; Figure 1).

Clinical intervention studies have demonstrated that increased dietary fiber intake elevates circulating SCFA levels and correlates with improvements in systolic blood pressure and inflammatory markers (Adiga et al., 2026; Iorra et al., 2026). Systematic reviews and meta-analyses of dietary interventions targeting the gut microbiome have shown beneficial effects on blood pressure, lipid profiles, and inflammatory markers in CVD patients (Un and Buys, 2015). However, the precise biological roles of individual SCFA subtypes remain incompletely understood, with emerging evidence suggesting distinct and sometimes overlapping functions that warrant further mechanistic elucidation (Chulenbayeva et al., 2025; O'Riordan et al., 2022). The protective effects of SCFAs extend to modulation of immune responses, mitochondrial function, and oxidative stress pathways, positioning them as critical mediators in cardiovascular health (Shi et al., 2026; Kulkarni and Gaikwad, 2025).

Despite promising data, challenges remain in translating SCFA biology into targeted therapies. Variability in gut microbial composition, host genetics, and dietary patterns influence SCFA production and responsiveness. Moreover, the systemic bioavailability and tissue-specific actions of SCFAs require further characterization. Nonetheless, SCFAs represent a cornerstone of the gut-heart axis’s protective arm, with dietary modulation and microbiota-targeted interventions offering feasible strategies to harness their cardioprotective potential.

### 3.3 Bile acid metabolites and farnesoid X receptor/G protein-coupled bile acid receptor 1 signaling

The bile acids (BAs), synthesized in the liver as primary bile acids, undergo extensive microbial transformation in the gut through bile salt hydrolase activity and 7α-dehydroxylation, generating secondary bile acids that serve as tissue-specific signaling molecules within host metabolic networks (Al Samarraie et al., 2023; Adiga et al., 2026) Bile acids undergo enterohepatic circulation, cycling between the intestine and liver via the portal vein. Microbial modification of primary bile acids into secondary bile acids alters FXR and TGR5 signaling not only in the liver and intestine but also systemically, affecting lipid metabolism, glucose homeostasis, and vascular function. These secondary bile acids act as endogenous ligands for nuclear receptor FXR and membrane-bound receptor TGR5, orchestrating lipid and glucose homeostasis with significant cardiovascular implications (Table 2; Figure 1).

Activation of FXR in hepatic and intestinal tissues regulates cholesterol metabolism, triglyceride synthesis, and glucose tolerance. Notably, intestinal FXR inhibition has been shown to promote brown adipose tissue thermogenesis and improve insulin sensitivity, conferring cardiovascular protection (Adiga et al., 2026; Yu et al., 2026). TGR5 activation in vascular endothelial cells and macrophages induces nitric oxide production and suppresses pro-inflammatory cytokine transcription, thereby exerting vasoprotective and anti-inflammatory effects (Adiga et al., 2026; Yu et al., 2026).

Gut dysbiosis alters the composition of the bile acid pool, shifting the balance between primary and secondary bile acids, which correlates with atherosclerosis progression and metabolic syndrome development (Al Samarraie et al., 2023; Adiga et al., 2026). Cholecystectomy removes the gallbladder reservoir and alters enterohepatic circulation, resulting in continuous bile acid delivery to the intestine and changes in the composition of the bile acid pool. This may increase exposure to secondary bile acids and modify FXR and TGR5 signaling, with potential effects on lipid metabolism, glucose homeostasis, and vascular function (Bilson et al., 2024; Al Samarraie et al., 2023; Adiga et al., 2026). Clinical evidence remains mixed; some studies report an association between cholecystectomy and increased risk of metabolic syndrome, dyslipidemia, and cardiovascular events, whereas others do not confirm a direct causal relationship. Post-cholecystectomy patients may therefore merit monitoring of bile acid profiles and cardiovascular risk factors, and this represents an important area for future research (Bilson et al., 2024; Al Samarraie et al., 2023; Adiga et al., 2026). Therapeutic strategies targeting bile acid metabolism, including probiotic modulation of bile salt hydrolase activity and pharmacological FXR agonists or antagonists, are advancing from mechanistic studies toward clinical evaluation (Adiga et al., 2026; Yu et al., 2026). These interventions aim to restore bile acid homeostasis and receptor signaling to mitigate cardiovascular risk. However, the complexity of bile acid signaling, influenced by species-specific receptor affinities and tissue distribution, poses challenges for therapeutic translation. Future research integrating multi-omics approaches and precision microbiome editing is essential to delineate bile acid-mediated pathways and optimize interventions targeting the FXR/TGR5 axis in cardiovascular disease (Figure 1).

### 3.4 Tryptophan metabolites and cardiovascular regulation through the aryl hydrocarbon receptor pathway

Gut microbiota metabolize dietary tryptophan into a diverse array of indole derivatives, including indole-3-propionic acid (IPA), indole-3-aldehyde (IAld), indole-3-acetate (IAA), indole-3-lactic acid (ILA), indole-3-acetamide (IAM), and tryptamine (Adiga et al., 2026; Yu et al., 2026; Table 2; Figure 1). These metabolites serve as endogenous ligands for the aryl hydrocarbon receptor (AhR), a ligand-activated transcription factor expressed in intestinal epithelial cells, immune cells, vascular endothelial cells, and smooth muscle cells (Adiga et al., 2026; Yu et al., 2026). AhR activation modulates gene expression programs involved in barrier function, immune homeostasis, and vascular tone (Adiga et al., 2026). For example, IAld promotes IL-22 production and supports mucosal barrier integrity, whereas IPA has been shown to reduce intestinal permeability and systemic endotoxemia (Adiga et al., 2026; Yu et al., 2026). IAA and ILA can also activate AhR, but their cardiovascular effects depend on context, dose, and tissue specificity (Adiga et al., 2026; Yu et al., 2026). The cardiovascular actions of AhR signaling are biphasic. Moderate, transient AhR activation maintains intestinal immune equilibrium, limits NF-κB-driven vascular inflammation, and supports endothelial function (Adiga et al., 2026). In contrast, chronic or excessive AhR activation has been implicated in vascular calcification, endothelial dysfunction, smooth muscle cell proliferation, and aneurysm formation (Adiga et al., 2026; Yu et al., 2026). This duality is influenced by ligand affinity, microbial composition, host genetics, and the local tissue microenvironment (Adiga et al., 2026; Yu et al., 2026). For instance, low-dose IAld is protective in models of endotoxemia and atherosclerosis, whereas sustained activation by other indole derivatives may promote vascular pathology (Adiga et al., 2026; Yu et al., 2026). Therapeutically, targeting the tryptophan–AhR axis requires precise modulation rather than simple agonism or antagonism (Adiga et al., 2026; Yu et al., 2026). Strategies include dietary tryptophan manipulation, probiotic strains that produce specific indole derivatives, and small-molecule AhR modulators with tissue-selective effects (Adiga et al., 2026; Yu et al., 2026). However, species-specific differences in AhR ligand affinity and tissue distribution pose challenges for translation (Adiga et al., 2026; Yu et al., 2026). Future studies should define which indole metabolites and AhR signaling patterns are protective versus harmful in specific cardiovascular disease stages (Adiga et al., 2026; Yu et al., 2026).

### 3.5 Intermediate metabolites of trimethylamine generation and potential intervention windows

Trimethylamine (TMA), the immediate precursor of TMAO, is produced by microbial TMA lyases from dietary choline, L-carnitine, and betaine. TMA possesses intrinsic biological activities, including osmotic regulation and protein stabilization, and circulating TMA may exert cardiovascular effects independent of TMAO, although these mechanisms remain underexplored (Zhen et al., 2023). Targeting microbial TMA lyases with non-bactericidal inhibitors, such as 3,3-dimethyl-1-butanol (DMB), a structural analog of choline, offers a strategy to reduce TMA and subsequent TMAO production without disrupting beneficial microbiota or inducing resistance (Zhen et al., 2023; Jia et al., 2019). DMB has demonstrated efficacy in animal models to attenuate atherosclerosis (Zhen et al., 2023). However, the long-term safety, pharmacokinetics, and clinical efficacy of such interventions require validation in large randomized controlled trials before clinical application (Hou et al., 2026; Chang et al., 2021).

### 3.6 Emerging metabolites and prospects for uncharacterized molecule discovery

Emerging metabolites include indole-3-propionic acid, imidazole propionate, p-cresol sulfate, and phenylacetylglutamine (PAGln). PAGln is a gut microbiota-derived metabolite of phenylalanine (Yu et al., 2026; Song et al., 2024; Romano et al., 2023). It enhances platelet integrin activation and thrombotic potential, while imidazole propionate and p-cresol sulfate have been associated with cardiometabolic phenotypes (Adiga et al., 2026; Yu et al., 2026). Microbial conversion of phenylalanine to phenylacetic acid, followed by host hepatic conjugation with glutamine, yields PAGln (Song et al., 2024; Romano et al., 2023). Mechanistically, PAGln acts as a ligand for adrenergic receptors, specifically α2A- and β2-adrenergic receptors (ADRA2A and ADRB2), on platelets and other cardiovascular cells (Song et al., 2024; Romano et al., 2023). Activation of these G protein-coupled receptors triggers downstream signaling, including intracellular Ca^2+^ mobilization and cAMP/PKA modulation, thereby enhancing platelet integrin αIIbβ3 activation, granule secretion, and thrombus formation (Song et al., 2024; Romano et al., 2023). In heart failure, PAGln levels correlate with disease severity and adverse outcomes, possibly through adrenergic modulation of cardiomyocyte and endothelial function (Romano et al., 2023). These mechanisms position PAGln as both a biomarker and a potential therapeutic target, with adrenergic receptor antagonists or microbial phenylalanine metabolism inhibitors as candidate interventions (Yu et al., 2026; Song et al., 2024; Romano et al., 2023).

The future of this field hinges on improving analytical sensitivity and expanding standard compound libraries to enable high-throughput identification and functional validation of candidate metabolites. Drugs can substantially reshape the gut microbiota and its metabolites, and the microbiota can in turn modify drug metabolism and toxicity (Karballaei Mirzahosseini et al., 2025). Antibiotics reduce microbial diversity and can lower TMAO and SCFA production, whereas metformin, statins, proton pump inhibitors, and antihypertensives have variable effects on microbial composition and metabolite profiles. Microbial enzymes can also metabolize cardiovascular drugs, altering their bioavailability and adverse effect profiles. These bidirectional interactions are clinically relevant because they may influence TMAO levels, SCFA availability, and response to cardiovascular therapy. A better understanding of drug–microbiota interactions is therefore essential for optimizing cardiovascular pharmacotherapy and for predicting inter-individual responses to microbiome-targeted interventions (Karballaei Mirzahosseini et al., 2025). Integrative multi-omics and systems biology approaches will be essential to elucidate the complex metabolic networks linking gut microbiota to cardiovascular disease, facilitating the development of novel biomarkers and therapeutic targets.

In summary, microbial metabolites exhibit a dualistic influence on cardiovascular physiology and pathology, with some promoting disease progression while others confer protection. Understanding these complex interactions provides a foundation for innovative diagnostic and therapeutic strategies targeting the gut-heart axis.

## 4 Gut microbiota-associated biomarkers in cardiovascular diseases, from single molecules to multidimensional models

### 4.1 Clinical evidence of microbial metabolites as diagnostic and prognostic biomarkers

The clinical utility of gut microbiota-derived metabolites as biomarkers in cardiovascular diseases has garnered substantial attention, particularly focusing on trimethylamine N-oxide, phenylacetylglutamine, and markers of gut permeability such as tight junction proteins. In acute coronary syndrome (ACS) patients, elevated plasma levels of TMAO and PAGln have demonstrated incremental prognostic value for both short-term and long-term adverse cardiovascular outcomes, surpassing traditional biomarkers like high-sensitivity cardiac troponin and C-reactive protein. TMAO, produced via hepatic oxidation of trimethylamine generated by gut bacteria metabolizing dietary choline, carnitine, and betaine, has been implicated in promoting atherosclerosis through mechanisms involving platelet hyperreactivity and vascular inflammation. Recent methodological advances, including capillary electrophoresis-based assays, have improved TMA quantification, facilitating its potential clinical application as a novel biomarker for CVD risk stratification (Li et al., 2023; Konop et al., 2021; Zhang et al., 2023). Similarly, PAGln, a gut microbiota-dependent metabolite derived from phenylalanine, correlates with heart failure severity and adverse outcomes, likely through modulation of adrenergic receptor signaling and platelet function, underscoring its prognostic significance beyond conventional markers (Song et al., 2024; Romano et al., 2023).

Conversely, SCFAs, especially butyrate, along with secondary bile acids and specific indole derivatives, exhibit decreased circulating levels in patients with stable coronary heart disease and heart failure, suggesting their protective roles. SCFAs, products of microbial fermentation of dietary fibers, contribute to maintaining gut barrier integrity, modulating immune responses, and exerting anti-inflammatory effects, which collectively mitigate cardiovascular risk. Secondary bile acids, generated by microbial biotransformation of primary bile acids, influence lipid metabolism and vascular function, while indole derivatives derived from tryptophan metabolism modulate endothelial function and inflammation (Table 3). The observed reductions in these metabolites in CVD patients highlight their dual potential as biomarkers and therapeutic targets (Al Samarraie et al., 2023; Wen et al., 2022; Liu et al., 2024).

**Table 3:** Gut microbiota-associated biomarkers in CVD: evidence level and dynamic vs. static features.

Biomarker	Type	Evidence level	Dynamic/Static	Clinical potential	References
TMAO	Metabolite	Large prospective cohorts/meta-analyses	Dynamic; diet- and renal function-dependent	Risk stratification, prognosis	Li et al. (2023), Zhen et al. (2023), and Sanchez-Gimenez et al. (2022)
PAGln	Metabolite	Clinical association; emerging mechanistic	Dynamic	Heart failure prognosis, thrombosis	Song et al. (2024) and Romano et al. (2023)
SCFAs	Metabolite	Clinical intervention and association	Dynamic; diet-dependent	Protective biomarker, therapeutic target	Al Samarraie et al. (2023) and Adiga et al. (2026)
Secondary bile acids	Metabolite	Mechanistic and clinical association	Dynamic; enterohepatic cycling	Metabolic and cardiovascular risk	Bilson et al. (2024), Al Samarraie et al. (2023), and Adiga et al. (2026)
Indole derivatives	Metabolite	Preclinical/mechanistic	Dynamic	Immune/vascular modulation	Adiga et al. (2026) and Yu et al. (2026)
cutC/cutD genes	Functional gene	Cross-cohort consistency	Relatively stable	TMA/TMAO production capacity	Li et al. (2023) and Al Samarraie et al. (2023)
7α-dehydroxylase	Functional gene	Mechanistic	Relatively stable	Bile acid transformation	Al Samarraie et al. (2023) and Adiga et al. (2026)
Zonulin/LPS	Barrier markers	Clinical association	Dynamic	Barrier dysfunction, inflammation	Chen et al. (2026) and Shi et al. (2026)
Microbial composition	Community	Variable across cohorts	Dynamic	Limited	Kim et al. (2022) and Liao et al. (2023)

### 4.2 Exploration of microbial composition and functional gene biomarkers

Beyond metabolites, gut microbial community composition and functional gene profiles have been investigated as biomarkers for cardiovascular diseases. Specific taxonomic ratios, such as the Streptococcaceae to Bacteroidales ratio, and diversity indices (α- and β-diversity) have been employed to distinguish acute myocardial infarction (AMI) patients from non-cardiovascular controls. However, these compositional markers often exhibit limited robustness across cohorts, reflecting interindividual variability and environmental influences (Kim et al., 2022; Liao et al., 2023). This limitation has shifted focus toward functional gene markers, which may offer greater stability and mechanistic insight. For instance, genes encoding enzymes critical for TMA production and bile acid 7α-dehydroxylase have been quantified, revealing stronger cross-individual consistency and direct links to pathogenic pathways in CVD (Li et al., 2023; Al Samarraie et al., 2023).

Recent advances in fecal metagenomics have enabled the construction of integrated microbial risk scores that incorporate both taxonomic and functional gene abundance data. Exploratory studies demonstrate that incorporating gut microbiome-derived risk scores into traditional cardiovascular risk models significantly improves net reclassification indices (NRI), enhancing predictive accuracy for adverse cardiovascular events. These findings extend the clinical utility of microbial biomarkers from mere association to actionable risk stratification tools, potentially guiding personalized prevention and treatment strategies (Kim et al., 2022; Warmbrunn et al., 2024; Table 3).

### 4.3 Novel risk stratification strategies based on combined biomarker panels

Emerging multidimensional models integrating host genetic risk scores, circulating metabolite biomarkers, and gut microbiome metagenomic data have shown superior predictive performance in cardiovascular disease risk stratification. Such integrative approaches enable identification of intermediate-risk individuals who are not adequately classified by traditional risk factors alone, thereby refining clinical decision-making (Jin et al., 2025; Lu et al., 2026). In heart failure management, inclusion of gut microbiota-derived biomarkers has opened new avenues for personalized medicine. For example, microbial markers have been incorporated into assessments of diuretic responsiveness, statin efficacy, and anticoagulant bleeding risk, offering a microbiome-guided dimension to pharmacotherapy optimization (Romano et al., 2023; Wen et al., 2022) However, the development of these complex multi-omics models necessitates rigorous internal and external validation to ensure generalizability. Confounding factors such as age, sex, and concomitant medications, particularly antihypertensives, lipid-lowering agents, and proton pump inhibitors, must be carefully accounted for during model construction, given their potential to alter gut microbiota composition and metabolite profiles (Table 3). Addressing these variables is critical to avoid spurious associations and to enhance the clinical applicability of microbiome-informed risk stratification tools (Warmbrunn et al., 2024; Sharma et al., 2025).

### 4.4 Challenges and standardization needs for biomarker translation from research to clinical practice

The translation of gut microbiota-associated biomarkers into clinical practice is hindered by several challenges. Globally, prospective multi-ethnic cohort studies lack standardized protocols for fecal and plasma sample collection, transportation, and storage, leading to batch effects and variability in sequencing and metabolite quantification data. This inconsistency undermines reproducibility and comparability across studies, impeding biomarker validation and regulatory approval (Warmbrunn et al., 2024; Gao et al., 2025; Table 3).

Furthermore, the intrinsic biological variability of gut microbiota markers within individuals, influenced by dietary intake, circadian rhythms, and seasonal changes, remains insufficiently quantified. This variability complicates the establishment of reference intervals and limits the reliability of single time-point measurements. Static single-time-point measurements may misclassify individuals because TMAO, SCFAs, bile acids, and other microbial metabolites fluctuate with diet, circadian rhythm, medication, disease stage, and seasonal changes. In contrast, dynamic biomarkers capture changes over time and can reflect treatment response, disease progression, or intervention effects. Repeated sampling, longitudinal cohorts, and wearable or metabolic sensors may better define individual baseline variability and improve risk stratification. Future studies should therefore distinguish static from dynamic biomarkers and establish standardized sampling windows and analytical protocols. Systematic investigations are required to characterize these fluctuations and to define standardized sampling windows and conditions to enhance biomarker stability (Warmbrunn et al., 2024; Wang et al., 2024).

On the regulatory front, there is an urgent need to develop clinical evaluation guidelines and quality control frameworks tailored to microbiota-related *in vitro* diagnostic products. Such frameworks should address assay validation, analytical performance, and clinical utility to support large-scale implementation of microbiome-based biomarkers in cardiovascular disease management. Establishing these standards will facilitate regulatory approval processes and promote integration into clinical decision pathways, ultimately improving patient outcomes (Warmbrunn et al., 2024).

## 5 Application and integration strategies of multi-omics tools in gut-heart axis research

### 5.1 Metagenomics and genome-wide association analysis

Shotgun metagenomic sequencing has revolutionized gut-heart axis research by providing high-resolution insights into microbial communities at species, strain, and gene-function levels, surpassing the taxonomic limitations of 16S rRNA amplicon sequencing. Unlike 16S rRNA sequencing, which primarily profiles bacterial taxonomy at genus or higher levels, shotgun metagenomics captures the entire microbial DNA content, enabling comprehensive functional annotation of microbial genes and pathways relevant to cardiovascular disease pathogenesis. This mechanistic depth is critical for elucidating how specific microbial functions influence host cardiovascular physiology and pathology. The development of metagenome-assembled genomes further enhances this capability by reconstructing near-complete genomes of uncultured or novel microbial species directly from complex gut samples. MAGs enable functional annotation even in the absence of reference genomes, facilitating discovery of previously uncharacterized microbial taxa and their metabolic potentials that may contribute to heart disease. For example, MAG-based analyses have identified novel microbial genes and pathways associated with coronary atherosclerosis and hypertension, expanding the repertoire of gut microbial factors implicated in CVD (Table 4; Figure 2). However, genome-wide association studies (GWAS) linking microbial genes to cardiovascular phenotypes require cautious interpretation due to confounding factors such as horizontal gene transfer, population stratification, and reverse causality. Large, well-controlled cohorts with rigorous statistical models are essential to disentangle causality from correlation. Integrating metagenomic data with host genomic and clinical information can further refine associations and identify microbial genetic determinants that modulate cardiovascular risk. Collectively, shotgun metagenomics and MAGs provide a powerful framework for mechanistic exploration of the gut microbiome’s role in cardiovascular health and disease, enabling identification of microbial biomarkers and therapeutic targets with unprecedented resolution (Mylavarapu et al., 2026; Edpuganti et al., 2025; Chen et al., 2026).

**Table 4:** Multi-omics approaches applied to the gut–heart axis: strengths, limitations, and key applications.

Approach	Strengths	Limitations	Key applications in gut–heart axis	References
Metagenomics/shotgun sequencing/MAGs	Species-, strain-, and gene-level resolution; functional annotation; MAGs recover uncultured taxa	Confounding by horizontal gene transfer, population stratification, reverse causality; requires large cohorts	Identify microbial genes/pathways in atherosclerosis and hypertension; discover biomarkers and targets	Mylavarapu et al. (2026), Edpuganti et al. (2025), and Chen et al. (2026)
Metabolomics (LC–MS, untargeted, isotope tracing)	Detects thousands of metabolites; distinguishes microbial, host, and co-metabolites; dynamic flux analysis	Standardization, batch effects, biological variability, annotation challenges	TMAO, SCFAs, bile acids, indole derivatives; biomarker discovery; mechanistic flux	Orjichukwu et al. (2025), Scisciola et al. (2026), and Valenbreder et al. (2023)
Proteomics/metaproteomics	Measures functional protein effectors; overcomes mRNA–protein mismatch; captures host response	Technical complexity, low-abundance proteins, sample processing	Map active microbial enzymes (SCFA/TMA); host ECM and complement proteins; functional networks	Chen et al. (2026) and Tang et al. (2026)
Single-cell/spatial omics	Resolves cellular heterogeneity and tissue-specific responses; preserves spatial context	Cost, dissociation bias, limited throughput, need for validation	Atherosclerotic plaque, myocardium, intestinal mucosa; cell-type-specific targets	Chen et al. (2026) and Tang et al. (2026)
Bioinformatics integration	Integrates heterogeneous data; identifies co-varying modules; supports causal inference	Lack of standardized annotation, pipelines, and interoperable repositories; overfitting risk	Multi-omics biomarker signatures; mechanism-based models	Chen et al. (2026) and Zeng et al. (2026)
Systems pharmacology/AI	Predicts drug–microbiota interactions; supports personalized therapy; identifies non-responders	Cost, reporting time, interpretability, clinical validation	Precision cardiovascular medicine; treatment monitoring; engineered strain optimization	Chen et al. (2026), Chandra et al. (2026), Moshref-Javadi et al. (2026), and Tanaka and Vécsei (2025)

**Figure 2:**
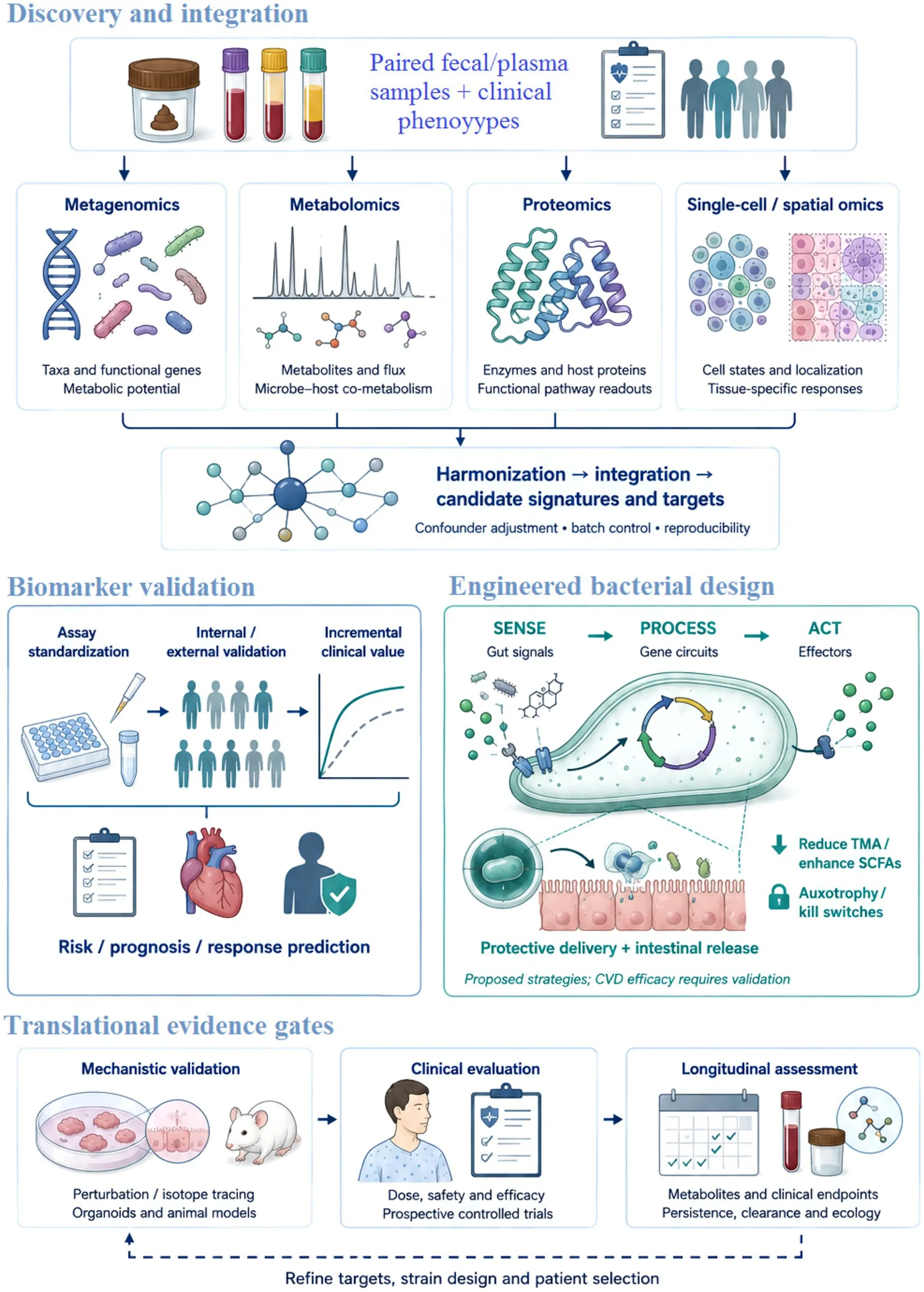
The proposed translational framework linking multi-omics discovery and biomarker validation to engineered bacterial design and clinical evaluation. This figure outlines the workflow from paired fecal/plasma sampling and multi-omics integration to biomarker validation, engineered bacterial design, and translational evidence gates.

### 5.2 Metabolomics in identifying microbiota-host co-metabolites

Metabolomics, particularly non-targeted liquid chromatography-mass spectrometry, serves as a pivotal tool for characterizing the complex chemical dialogue between gut microbiota and host in the gut-heart axis. This approach enables simultaneous detection and quantification of thousands of metabolites in fecal and plasma samples, providing a comprehensive snapshot of microbial and host metabolic activities. By profiling paired plasma and fecal metabolomes, researchers can distinguish metabolites directly produced by gut microbes, those modified by host metabolism, and molecules synthesized through host-microbe co-metabolic pathways. Such differentiation is crucial for understanding the origin and systemic impact of metabolites implicated in cardiovascular diseases, such as TMAO, SCFAs, bile acids, and indole derivatives. Isotope tracing metabolomics further refines this understanding by dynamically tracking the metabolic fate and inter-organ transport of key microbial metabolites (Table 4; Figure 2). For instance, isotope-labeled precursors have elucidated the biosynthesis and systemic distribution of TMAO and SCFAs, revealing their roles in modulating endothelial function, inflammation, and myocardial metabolism. These dynamic flux analyses provide indispensable kinetic and mechanistic evidence beyond static metabolite abundance, informing targeted interventions to modulate metabolite production or action. Overall, metabolomics offers a direct and sensitive strategy to decode the chemical mediators of the gut-heart axis, enabling biomarker discovery and mechanistic insights into microbiota-driven cardiovascular pathophysiology (Orjichukwu et al., 2025; Scisciola et al., 2026; Valenbreder et al., 2023).

### 5.3 Proteomics and inference of metabolic pathway activity

Proteomics complements genomic and metabolomic data by directly measuring the functional protein effectors expressed by gut microbiota and host tissues, thereby providing a more accurate depiction of active metabolic pathways within the gut-heart axis. Fecal metaproteomics allows detection of microbial enzymes and functional proteins, overcoming limitations of transcriptomic analyses where mRNA abundance may not correlate with protein activity due to post-transcriptional regulation. This enables precise mapping of microbial metabolic activities influencing cardiovascular health, such as enzymes involved in SCFA production or TMA synthesis. Concurrently, quantitative plasma proteomics can monitor host protein responses to microbial metabolites, including extracellular matrix remodeling proteins and complement cascade components implicated in cardiac fibrosis and inflammation. These protein-level data facilitate construction of downstream functional networks linking microbial metabolite exposure to host pathophysiological responses. Emerging single-cell proteomics technologies further enhance resolution by enabling characterization of immune cell subtype-specific cytokine secretion profiles in cardiovascular tissues. This fine-scale cellular phenotyping is critical for dissecting immune mechanisms underlying gut-heart axis interactions, such as macrophage polarization and T cell responses modulated by microbial metabolites. As single-cell proteomics matures, it promises to provide unprecedented insights into cell-type-specific functional states and intercellular communication within cardiovascular microenvironments influenced by gut microbiota (Table 4; Figure 2). Thus, proteomics bridges the gap between microbial gene potential and host functional outcomes, advancing mechanistic understanding and biomarker development in gut-heart axis research (Chen et al., 2026; Tang et al., 2026).

### 5.4 Single-cell sequencing and spatial omics reveal tissue-specific responses

Single-cell RNA sequencing (scRNA-seq) has become an indispensable tool for resolving the cellular heterogeneity and tissue-specific responses within the gut-heart axis. Applied to atherosclerotic plaques, myocardium, and intestinal mucosa, scRNA-seq delineates distinct immune and non-immune cell populations and their transcriptional states in response to microbial metabolites. This high-resolution profiling uncovers cell-type-specific receptor expression, signaling pathways, and effector functions that mediate microbial influence on cardiovascular pathology. Complementing scRNA-seq, spatial transcriptomics preserves tissue architecture, enabling mapping of metabolite-responsive gene expression modules to precise anatomical locations within diseased vascular or cardiac tissues. This spatial context is vital for understanding microenvironmental niches where gut-derived signals modulate local inflammation, fibrosis, or endothelial dysfunction. Furthermore, advanced *in vitro* models such as organoids and microfluidic organ-on-chip systems provide controlled platforms for mechanistic validation of single-cell findings. These models recapitulate human cardiovascular tissue complexity and allow causal interrogation of microbial metabolite effects on cellular transcriptional programs. Together, single-cell and spatial omics technologies offer transformative insights into the cellular and spatial dynamics of gut-heart communication, facilitating identification of novel therapeutic targets and biomarkers with tissue and cell-type specificity (Chen et al., 2026; Tang et al., 2026; Table 4; Figure 2).

### 5.5 Bioinformatics integration frameworks for multi-omics data

The integration of multi-omics datasets in gut-heart axis research necessitates robust bioinformatics frameworks capable of synthesizing heterogeneous data types into coherent biological insights. Statistical correlation network approaches, such as sparse canonical correlation analysis and multi-factor analysis, enable dimensionality reduction and identification of co-varying modules linking microbial taxa, gene functions, metabolite abundances, and protein expression with cardiovascular phenotypes. These methods facilitate discovery of multi-layered biomarker signatures and functional modules underpinning disease processes. Mechanism-based integration frameworks advance beyond correlation by incorporating metabolic flux models and host transcriptional regulatory networks. Bayesian networks and causal inference algorithms enhance predictive power by modeling directional relationships and disentangling confounding effects, thus improving reliability of multi-omics-derived hypotheses. A critical bottleneck in multi-omics integration is the lack of standardized data annotation, harmonized computational pipelines, and interoperable public repositories (Table 4; Figure 2). Containerized workflows and unified metadata standards are essential to enable reproducibility, cross-study comparisons, and meta-analyses that increase statistical power and reduce batch effects. Investment in these infrastructure components will accelerate the translation of multi-omics data into actionable insights for gut-heart axis biology and clinical applications (Chen et al., 2026; Zeng et al., 2026).

### 5.6 Systems pharmacology applications and potential clinical decision support

Multi-omics integration within a systems pharmacology framework offers promising avenues for personalized cardiovascular medicine by modeling patient-specific gut microbiota-metabolite-drug interaction networks. These models can predict individual variability in drug response and adverse effect risk mediated by microbial metabolism of cardiovascular agents such as antiplatelets, statins, and anticoagulants. Dynamic monitoring of multi-omics profiles during treatment enables real-time assessment of therapeutic impact on gut microbial ecology and metabolite production, facilitating proactive regimen adjustments to optimize cardiovascular protection. Artificial intelligence (AI)-driven algorithms that integrate multi-omics data are beginning to identify microbial metabolic signatures predictive of clinical benefit in phase II trials, marking a step toward precision microbiome-informed therapy. Recent advances in machine learning and deep learning have further expanded the analytical toolkit for gut–heart axis research. Random forest and XGBoost can be used for feature selection and risk prediction from multi-omics data. Deep learning and multimodal fusion models can integrate metagenomic, metabolomic, proteomic, and imaging data to predict treatment response. Graph neural networks are particularly suitable for encoding microbial interaction networks and metabolic pathway topologies, thereby improving interpretability and predictive accuracy. These approaches may help identify responder signatures and support personalized microbiome-targeted cardiovascular interventions.

However, challenges remain in reducing assay costs, shortening reporting times, and enhancing interpretability to enable routine clinical implementation (Table 4; Figure 2). From a practical perspective, the choice of multi-omics approach should be guided by the specific research question. Shotgun metagenomics and metagenome-assembled genomes are most appropriate for identifying microbial taxa, genes, and pathways. Untargeted or targeted metabolomics, ideally combined with isotope tracing, is suited to discovering microbial–host co-metabolites and dynamic flux. Metaproteomics and plasma proteomics can define active microbial enzymes and host protein responses. Single-cell and spatial omics are preferable when cell-type-specific or tissue-localized responses are of interest. For data integration, sparse canonical correlation analysis, multi-factor analysis, and Bayesian networks can identify co-varying modules and causal hypotheses. Finally, machine learning, deep learning, and graph neural networks are useful for prediction, responder identification, and strain optimization. Continued innovation in multi-omics technologies, computational methods, and clinical validation will be critical to realize the full potential of gut-heart axis-informed systems pharmacology for guiding individualized cardiovascular treatment decisions (Chen et al., 2026; Chandra et al., 2026).

## 6 Frontier applications of engineered bacterial delivery systems in gut-heart axis regulation

### 6.1 Fundamental logic of engineered bacteria design and synthetic biology tools

The design of engineered bacteria for modulating the gut-heart axis follows a cyclical framework encompassing detection, computation, and execution phases. Initially, the sensing module is tailored to detect disease-associated molecular signals within the gut environment, such as metabolites or inflammatory markers relevant to cardiovascular pathophysiology. Upon detection, the computational module processes these inputs through engineered genetic circuits that perform logical operations and signal amplification, ensuring precise and robust responses. Finally, the execution module triggers the production and secretion of therapeutic effector molecules, such as enzymes or peptides, that can modulate host metabolism or immune responses to ameliorate cardiovascular conditions. This modular design is underpinned by advanced synthetic biology tools, notably the CRISPR-Cas system, which enables precise, low off-target genome editing of bacterial strains (Fan et al., 2023). The availability of standardized genetic parts, such as promoters, ribosome binding sites, and terminators, facilitates the assembly of complex and stable gene circuits (Table 5; Figure 3), allowing for predictable and tunable gene expression (Mylavarapu et al., 2026). The modularity also permits independent optimization and replacement of sensing and output components, enabling the construction of bespoke bacterial strains targeting specific cardiovascular metabolic pathways. Such a design philosophy enhances adaptability and scalability, critical for translating engineered bacteria into clinical applications targeting the gut-heart axis (Chen et al., 2026). Representative genetic circuits developed for engineered bacteria include AND gates that integrate multiple disease-associated signals, toggle switches that provide bistable memory, quorum-sensing modules that coordinate population-level responses, CRISPRi-based circuits for tunable gene repression, and negative feedback loops that stabilize therapeutic output. These circuits have been adapted for gut-based sensing and therapeutic delivery, and they provide a foundation for designing strains that respond precisely to cardiovascular-relevant signals.

**Table 5:** Preclinical studies of engineered bacteria in CVD models: organism, modification, model, outcomes, and references.

Organism	Modification	Model	Outcomes	References
Engineered TMAO-silencing bacterial strain	TMA-silencing genetic circuit; inhibition of microbial TMA production; secretion of competitive peptides against TMA lyase	ApoE−/− mice, atherosclerosis	Reduced aortic root plaque burden; decreased plasma pro-inflammatory cytokines; reduced macrophage infiltration; lower circulating TMAO; attenuated atherosclerosis progression	Zhen et al. (2023)
Butyrate-producing engineered bacteria	Introduction of butyrate biosynthetic gene cluster into non-butyrate-producing strain	Myocardial ischemia–reperfusion injury model	Enhanced cardiac gap junction protein expression; reduced infarct size; improved morphological and functional cardiac outcomes	Zeng et al. (2026)

**Figure 3:**
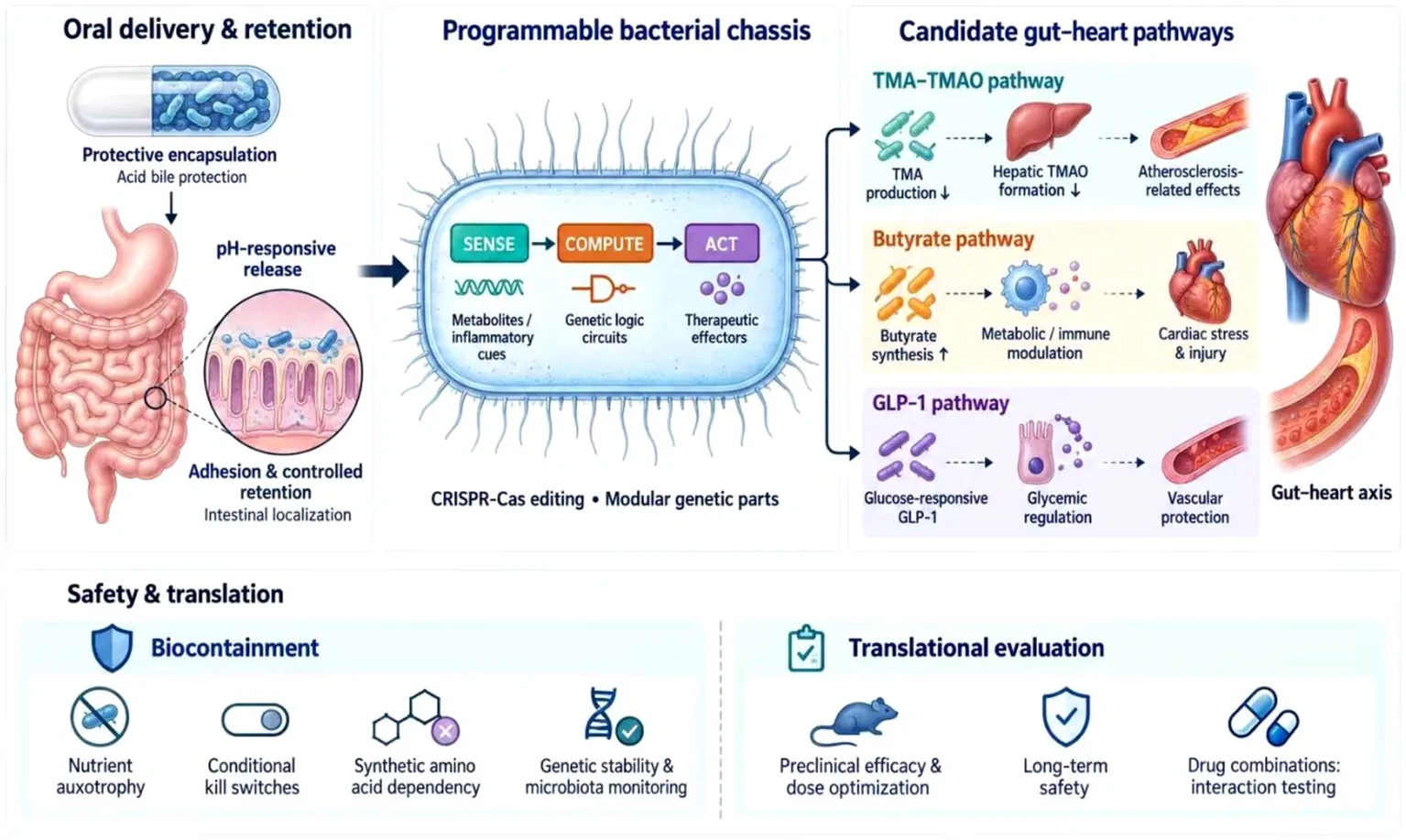
Engineered bacterial sensing, computation, execution, delivery, colonization, biocontainment, and preclinical outcomes.

### 6.2 Gene circuits for intestinal localization and metabolic pathway reconstruction

Engineered bacteria have been successfully programmed to function within the anaerobic environment of the gut to modulate key metabolic pathways implicated in cardiovascular disease. For example, strains expressing TMA lyase inhibitors have been designed to secrete competitive peptides that reduce TMA production in the intestinal lumen. Since TMA is converted in the liver to TMAO, a pro-atherogenic metabolite, this strategy effectively lowers circulating TMAO levels and attenuates atherosclerosis progression (Zhen et al., 2023). Additionally, metabolic engineering has introduced gene clusters encoding SCFA biosynthetic enzymes into non-butyrate-producing strains, enabling sustained *in situ* butyrate synthesis. In anthracycline-induced cardiomyopathy models, this approach significantly mitigated oxidative stress and apoptosis in cardiac tissue, highlighting the cardioprotective potential of engineered microbial metabolites (Chulenbayeva et al., 2025). Moreover, glucose-responsive engineered bacteria have been developed by integrating blood glucose-sensitive promoters controlling the secretion of glucagon-like peptide-1 (GLP-1). This coupling allows dynamic GLP-1 release in response to intestinal glucose levels, providing simultaneous glycemic control and vascular protection in diabetic cardiovascular disease models (Zeng et al., 2026). These advances demonstrate the feasibility of reconstructing metabolic pathways and gene circuits within engineered bacteria to achieve targeted, context-dependent therapeutic effects in the gut-heart axis (Table 5; Figure 3).

### 6.3 Optimization of delivery vectors and colonization strategies

Effective oral delivery of engineered bacteria necessitates overcoming multiple gastrointestinal barriers, including gastric acidity, bile salts, and the mucosal layer. To address these challenges, various delivery vehicles have been developed, such as natural extracellular polysaccharide coatings, liposomal encapsulation, and pH-responsive hydrogel microcapsules (Yu et al., 2026). These carriers protect bacteria during transit and facilitate controlled release in the intestine. Furthermore, colonization efficiency is enhanced by incorporating adhesin modules or engineering strain-specific colonization factors that promote targeted retention in defined gut segments, such as the terminal ileum or proximal colon (Table 5; Figure 3). However, intestinal localization alone is insufficient for cardiac targeting. Engineered bacteria typically act as intestinal factories that release therapeutic molecules into the portal or systemic circulation. To achieve cardiac specificity, future strategies could combine region-specific colonization with inducible release of engineered outer membrane vesicles, nanocarrier conjugation, or cardiac-targeting ligands. Hypoxia-responsive promoters, inflammation-responsive circuits, and quorum-sensing modules may further restrict therapeutic release to diseased microenvironments. This spatial targeting extends the duration of therapeutic gene expression and functional activity within the host. Genetic enhancement of biofilm formation capacity also improves bacterial persistence in the competitive gut microbiota ecosystem (Adiga et al., 2026). However, this strategy requires careful balance to avoid excessive colonization that could trigger host immune responses or disrupt native microbial communities. Overall, optimizing delivery and colonization strategies is critical to maximize the therapeutic window and safety profile of engineered bacterial interventions in cardiovascular disease (Chandra et al., 2026). The duration for which engineered bacteria remain stable in the gut and continue to modulate cardiovascular metabolic pathways is highly variable and depends on the strain, genetic circuit design, delivery vehicle, colonization niche, diet, host immunity, antibiotic exposure, and native microbiota. In preclinical models, non-colonizing vehicles may persist for only hours to days, whereas engineered strains with adhesion modules or auxotrophic dependencies can colonize for days to weeks; long-term persistence beyond weeks is uncommon and raises safety concerns. Consequently, therapeutic effects on TMAO, SCFAs, bile acids, or GLP-1 may be transient and reversible, and sustained modulation often requires repeated administration, robust colonization, inducible circuits, and real-time monitoring of strain abundance and metabolite levels to define the duration of action and maintain efficacy. Long-term stability, plasmid maintenance, and escape rates should therefore be systematically evaluated, and biocontainment strategies should balance persistence with safety; long-term studies should also quantify colonization kinetics, escape rates, and effects on native microbiota (Fan et al., 2023; Adiga et al., 2026; Yu et al., 2026; Mylavarapu et al., 2026; Chen et al., 2026; Chandra et al., 2026). These challenges become even more pronounced at clinical scale, where manufacturing must address strain stability and plasmid maintenance during large-scale fermentation, lyophilization and storage, viable-cell quantification, potency assays, and batch-to-batch consistency under good manufacturing practice (GMP), as well as scalable downstream processing, quality control, and standardized release criteria. Addressing these manufacturing issues early will be critical for regulatory approval and clinical translation of engineered bacterial therapies.

### 6.4 Safety assessment and biocontainment mechanisms

Safety concerns surrounding engineered bacteria primarily involve uncontrolled proliferation and horizontal gene transfer, which could pose risks of dysbiosis or unintended genetic dissemination. To mitigate these issues, multiple biocontainment strategies have been integrated into bacterial designs. Nutritional auxotrophy, where engineered strains depend on externally supplied essential nutrients absent in the host environment, restricts survival outside controlled conditions. Toxin-antitoxin systems and conditional suicide switches provide fail-safe mechanisms that trigger bacterial self-elimination under predefined conditions, such as absence of specific inducers or environmental cues (Fan et al., 2023). Synthetic amino acid dependency further enhances containment by rendering bacteria inviable without supplementation of non-natural amino acids. Specific kill-switch systems include toxin–antitoxin modules such as hok/sok and MazEF, which mediate post-segregational killing or conditional cell death; CRISPR-Cas3-based self-targeting systems that induce controlled genome cleavage; nutritional auxotrophy, in which strains depend on externally supplied nutrients absent in the host; and synthetic amino acid dependency. These systems can be combined with inducible promoters to create fail-safe biocontainment, and their long-term stability and escape rates should be rigorously evaluated in preclinical models. Additionally, inducible control systems responsive to temperature or chemical inducers enable dynamic regulation of therapeutic protein expression, as validated in rodent models (Mylavarapu et al., 2026). Long-term safety evaluation also encompasses monitoring mutation rates, plasmid stability, and impacts on native microbiota structure to identify potential risks associated with chronic administration (Chen et al., 2026). These comprehensive safety and biocontainment frameworks are essential for advancing engineered bacteria toward clinical translation in cardiovascular therapeutics (Table 5; Figure 3).

### 6.5 Preclinical pharmacodynamic validation of engineered bacteria

Preclinical studies have demonstrated the therapeutic efficacy of engineered bacteria in cardiovascular disease models. In ApoE knockout mice, oral administration of TMAO-silencing engineered strains significantly reduced aortic root plaque burden and decreased plasma pro-inflammatory cytokines and macrophage infiltration, indicating potent anti-atherosclerotic effects (Zhen et al., 2023). Similarly, in myocardial ischemia–reperfusion injury models, butyrate-producing engineered bacteria enhanced expression of cardiac gap junction proteins and reduced infarct size, resulting in improved morphological and functional cardiac outcomes post-injury (Zeng et al., 2026). Pharmacological and engineered bacterial approaches to myocardial ischemia–reperfusion injury differ in onset, duration, and risk profile. Pharmacological thrombolysis, such as reteplase, can rapidly restore perfusion but carries systemic bleeding risk, as illustrated in a case of pulmonary embolism causing cardiac arrest during neurosurgery (Yousefi-Mazhin et al., 2024). In contrast, engineered bacteria can produce cardioprotective metabolites such as butyrate locally and sustainably, potentially reducing infarct size and adverse remodeling with a slower onset and more variable colonization. Combining rapid pharmacological reperfusion with sustained microbial cardioprotection may offer complementary benefits, but safety and drug–bacteria interactions must be carefully evaluated (Yousefi-Mazhin et al., 2024). These findings underscore the capacity of engineered microbial metabolites to modulate cardiac remodeling and inflammation. Sepsis is characterized by systemic inflammation, LPS translocation, and profound microbial dysbiosis, all of which can exacerbate cardiovascular injury (Zhernakova et al., 2016; Jie et al., 2017). Engineered bacteria could potentially be designed to neutralize LPS, deliver anti-inflammatory cytokines, or restore beneficial microbial functions in the septic host. However, sepsis is a high-risk condition with compromised barrier integrity and immune dysregulation, so engineered strains must be rigorously evaluated for biocontainment, antibiotic compatibility, and the risk of exacerbating inflammation. Pharmacokinetic and clinical studies in critically ill patients, such as those examining high-dose oral rifampicin and sepsis management in complex cases, provide important lessons for the safe development of engineered bacterial therapies in this setting (Karballaei-Mirzahosseini et al., 2022; Karballaei Mirzahosseini et al., 2025). While clinical trials of engineered bacteria have predominantly focused on metabolic and inherited metabolic disorders, cardiovascular applications remain in early stages. Establishing clear dose-escalation protocols and standardized endpoints will be critical for advancing engineered bacterial therapies targeting the gut-heart axis into human studies (Yu et al., 2026).

Combining engineered bacteria with cardiovascular drugs may not always produce synergistic benefits and could exacerbate adverse effects or drug interactions. Engineered strains may alter drug metabolism, gut permeability, vitamin K availability, and platelet function, potentially increasing bleeding risk in patients receiving anticoagulants or antiplatelet agents. A recent case of massive gastrointestinal bleeding associated with apixaban, diltiazem, and aspirin illustrates the serious consequences of complex drug interactions (Mirzahosseini et al., 2024). Therefore, co-administration of engineered bacteria with cardiovascular drugs requires rigorous pharmacokinetic and pharmacodynamic evaluation, pharmacovigilance, and careful patient selection. Clinical-scale manufacturing of engineered bacteria will require robust strain stability, scalable anaerobic fermentation, lyophilization or encapsulation, batch-to-batch consistency, and validated potency assays (Mylavarapu et al., 2026; Chen et al., 2026; Chandra et al., 2026).

### 6.6 Synergistic therapeutic strategies combining engineered bacteria and cardiovascular drugs

Combining engineered bacterial therapies with conventional cardiovascular drugs offers promising synergistic benefits. For instance, butyrate produced *in situ* by engineered bacteria can synergize with angiotensin-converting enzyme inhibitors (ACEIs) to attenuate angiotensin II-induced vascular inflammation while mitigating bradykinin accumulation-related side effects such as cough (Chandra et al., 2026). In antiplatelet therapy, engineered *Lactococcus lactis* strains secreting fibrinolytic enzymes like nattokinase or hirudin locally in the gut can be absorbed systemically to provide mild, sustained antithrombotic effects, potentially reducing bleeding risks associated with standard antiplatelet agents (Chandra et al., 2026). Preclinical animal studies assessing drug distribution and metabolic profiles are essential to ensure safety and efficacy of such combination regimens (Chen et al., 2026). These integrative approaches highlight the potential of engineered bacteria to complement and enhance existing cardiovascular pharmacotherapies through gut-mediated mechanisms (Figure 3).

## 7 Current challenges and future research directions

### 7.1 Translational dilemma of inter-species differences from animal models to humans

A critical challenge in gut-heart axis research lies in the translational gap between findings derived from rodent cardiovascular disease models and their applicability to human physiology. Many key microbial metabolic targets identified in rodent models, such as specific bile acid receptors and SCFA receptor subtypes, exhibit significant interspecies differences in expression levels, tissue distribution, and ligand affinity. For instance, bile acid receptor farnesoid X receptor and G-protein coupled bile acid receptor 1 (TGR5) have distinct expression patterns and functional roles across species, which complicates direct extrapolation of preclinical efficacy data to humans (Xu et al., 2021). Similarly, SCFA receptors such as GPR41 and GPR43 show variable expression and signaling dynamics between rodents and humans, influencing the downstream cardiovascular and metabolic effects of microbial metabolites. This species-specific variability necessitates cautious interpretation of preclinical data and underscores the importance of integrating complementary human-relevant models. To mitigate translational risks, preclinical pharmacodynamic and mechanistic findings should be corroborated with data from human *ex vivo* organ models, such as isolated human vascular or cardiac tissues, and advanced organoid culture systems that recapitulate human tissue architecture and cellular heterogeneity. Moreover, leveraging multi-omics datasets from well-characterized clinical cohorts can provide critical validation of microbial targets and pathways implicated in cardiovascular pathology. Another promising approach to bridge this gap involves the development of humanized microbiota mouse models, wherein germ-free mice are colonized with human gut microbiota to better mimic human microbial-host interactions. Embedding a broader spectrum of human immune cell populations into these models can further reduce systemic biases arising from interspecies immune-metabolic differences, thereby enhancing the physiological relevance of findings (Duan et al., 2026).

### 7.2 Intervention windows and personalized dynamic monitoring strategies for gut-heart axis modulation

The temporal dynamics of cardiovascular disease progression critically influence the responsiveness to gut microbiota-targeted interventions. Different pathological stages, from early endothelial dysfunction to advanced heart failure, exhibit variable sensitivity to microbial metabolites and their signaling pathways. For example, early-stage endothelial impairment may be more amenable to modulation of SCFA levels or bile acid signaling, whereas late-stage heart failure might require combinatorial approaches addressing systemic inflammation and metabolic derangements (Yang et al., 2021). Therefore, defining optimal intervention windows aligned with disease chronology is essential to maximize therapeutic efficacy. Currently, no live biotherapeutic product has been approved by the FDA for CVD. Early-phase clinical trials have explored dietary interventions, probiotics, and fecal microbiota transplantation for effects on TMAO, blood pressure, and inflammatory markers, but these studies remain small, short-term, and largely reliant on surrogate endpoints. This represents a major translational gap and supports the need for adequately powered, endpoint-driven trials. This necessitates stratification of patients based on disease stage and molecular phenotypes to tailor microbiota-directed therapies appropriately, whether for primary prevention, acute management, stabilization, or rehabilitation phases. To enable such precision, continuous and non-invasive monitoring platforms are indispensable. Integrating serial fecal sampling with wearable metabolic sensors capable of real-time detection of microbial metabolites or host physiological parameters can facilitate longitudinal tracking of gut-heart axis status in home settings. Such systems would allow dynamic adjustment of interventions in response to evolving biomarker profiles, thereby enhancing treatment personalization and adherence. Furthermore, inter-individual variability in baseline microbiota composition, dietary habits, and host genetic polymorphisms contributes to heterogeneous treatment responses. To address this, future randomized clinical trials should incorporate adaptive enrichment designs that enable identification and quantification of treatment effect heterogeneity. Embedding multi-omics profiling and machine learning algorithms within trial frameworks can facilitate the development of predictive models for individual responsiveness, ultimately guiding personalized microbiota-targeted cardiovascular therapies (Sublette et al., 2021). This approach promises to refine intervention timing and optimize outcomes by accounting for the complex interplay of host and microbial factors.

Diet should be considered an important adjunct to engineered bacterial therapies. Dietary substrates can shape the availability of precursors for beneficial microbial metabolites, reduce the production of harmful metabolites such as TMA, and influence the engraftment, persistence, and metabolic activity of engineered strains. For example, fiber-rich diets can promote SCFA production and may enhance the efficacy of butyrate-producing engineered bacteria, while reduced intake of choline, carnitine, and betaine may lower TMAO levels and complement TMA-lyase-targeting strategies. Personalized dietary recommendations, guided by microbiome and metabolite profiling, may therefore improve the effectiveness of engineered bacterial interventions and should be integrated into future clinical trial designs.

### 7.3 Prospects of artificial intelligence and multi-omics integration in gut-heart axis research

The complexity of the gut-heart axis, encompassing diverse microbial taxa, metabolic pathways, host immune responses, and clinical phenotypes, demands sophisticated analytical frameworks capable of integrating heterogeneous data modalities. Deep learning-based multimodal fusion models have emerged as powerful tools to jointly analyze metagenomic sequences, metabolomic profiles, gut barrier integrity markers, and imaging data. Such integrative modeling enables prediction of individual responses to microbiota-targeted interventions and facilitates early identification of non-responders, thereby informing clinical decision-making (Moshref-Javadi et al., 2026). Graph neural networks (GNNs) offer a particularly promising approach by encoding microbial species interaction networks alongside metabolic pathway topologies into structured representations. This allows capturing complex interdependencies and signaling cascades within the microbiome-host ecosystem, enhancing the interpretability and predictive accuracy of response models. Moreover, the design and optimization of engineered probiotic strains for cardiovascular targets can benefit from reinforcement learning-based automated platforms. These platforms iteratively refine genetic circuit designs to enhance strain performance, accelerating the discovery of high-efficacy microbial therapeutics tailored to modulate specific cardiovascular pathways (Tanaka and Vécsei, 2025). The convergence of AI-driven multi-omics integration and synthetic biology thus holds transformative potential for precision gut-heart axis interventions, enabling rapid translation from mechanistic insights to clinically viable engineered microbial therapeutics.

### 7.4 Ethical and regulatory considerations and patient acceptance

The clinical deployment of engineered bacteria as live biotherapeutic agents introduces novel regulatory and ethical challenges. Unlike conventional pharmaceuticals, these living drugs require new definitions within medical product regulatory frameworks to address their unique properties, including replication, genetic modification, and potential environmental impact. Regulatory agencies worldwide must develop adaptive governance models that balance innovation facilitation with stringent biosafety oversight, encompassing patent approval processes and clinical trial access (Duan et al., 2026). Patient acceptance is equally critical; comprehensive informed consent procedures should transparently communicate the composition of engineered strains, potential unintended clearance mechanisms, and long-term monitoring plans to build trust and ensure ethical compliance. Given the substantial geographic and ethnic variability in gut microbiota composition, international collaborative clinical studies must navigate regional ethical approvals while ensuring equitable sharing of microbial strain characterization data and therapeutic outcomes. This approach safeguards participant health rights and promotes fair distribution of benefits arising from microbiota-based therapies, fostering global harmonization and public confidence in this emerging therapeutic paradigm.

In the United States, live biotherapeutic products (LBPs) are regulated by the FDA Center for Biologics Evaluation and Research (CBER) as biological products, generally requiring an Investigational New Drug (IND) application under 21 CFR Part 312. Manufacturing must comply with current Good Manufacturing Practice (cGMP) under 21 CFR Parts 210 and 211. Relevant FDA guidance includes “Early Clinical Trials with Live Biotherapeutic Products: Chemistry, Manufacturing, and Control Information” (Meredith and Elliott, 1994). In Europe, LBPs are evaluated under the advanced therapy medicinal product (ATMP) framework by the European Medicines Agency (EMA), with additional national requirements (Schuessler-Lenz et al., 2023). These frameworks should be considered early in strain design and clinical development.

### 7.5 Key milestones for future clinical applications

Short-term clinical objectives should prioritize rigorous phase II trials to establish the safety and efficacy of single-strain probiotics or microbial metabolite modulators in well-defined cardiovascular subpopulations, such as patients with elevated TMAO levels and coronary artery disease. Concurrently, optimization of delivery formulations and compliance with regulatory quality standards are essential to facilitate clinical translation (Li et al., 2021). Mid-term goals involve advancing multi-strain engineered microbial consortia and fixed-composition products through clinical development pipelines, with stratified indications tailored to primary prevention, secondary prevention, or adjunctive pharmacotherapy contexts. This stratification aims to maximize reproducible clinical benefits across diverse cardiovascular phenotypes. Long-term aspirations encompass establishing closed-loop precision medicine frameworks that integrate gut microbiome multi-omics signatures with individual metabolic phenotypes to guide personalized nutrition and therapeutic regimens. Such integrative management pathways promise to revolutionize cardiovascular care by harnessing the full potential of microbiota-based interventions within precision health paradigms.

Clinical trials of microbiome-targeted interventions in CVD remain limited. A few small trials have evaluated probiotics, prebiotics, or TMAO-lowering strategies, but large-scale randomized controlled trials with hard cardiovascular endpoints are still lacking (Un and Buys, 2015). This represents a critical evidence gap and a key priority for future research.

To provide a more specific timeline for clinical translation, we propose short-term (0–2 years), medium-term (3–5 years), and long-term (5–10 years) milestones (Sethi et al., 2023; Warmbrunn et al., 2024; Chandra et al., 2026). Short-term: harmonize sample collection, metabolite assays, and metadata standards (Chang et al., 2021; Konop et al., 2021; Liao et al., 2023; Warmbrunn et al., 2024); establish multi-center biobanks (Warmbrunn et al., 2024); retrospectively validate candidate biomarkers in existing cohorts (Sanchez-Gimenez et al., 2022; Kim et al., 2022; Liao et al., 2023; Warmbrunn et al., 2024); form regulatory-science working groups for microbiome-based diagnostics (Warmbrunn et al., 2024; Chandra et al., 2026); and perform early health-economic modeling (Warmbrunn et al., 2024; Chandra et al., 2026). Medium-term: conduct prospective multi-ethnic cohort studies and biomarker-guided randomized controlled trials (RCTs) for TMAO-lowering, SCFA-promoting, or bile-acid-modulating interventions; initiate first-in-human trials of engineered bacterial delivery systems with strict biocontainment (Fan et al., 2023; Chen et al., 2026; Chandra et al., 2026); and refine AI-based predictive models for responder identification (Chen et al., 2026; Chandra et al., 2026; Moshref-Javadi et al., 2026; Tanaka and Vécsei, 2025). Long-term: complete phase III RCTs and regulatory approvals (Adiga et al., 2026; Yu et al., 2026; Chandra et al., 2026); integrate validated microbiome biomarkers into cardiovascular risk scores and clinical guidelines (Kim et al., 2022; Warmbrunn et al., 2024; Jin et al., 2025; Lu et al., 2026; Sharma et al., 2025); implement personalized, dynamically monitored microbiome therapies (Wen et al., 2022; Chen et al., 2026; Chandra et al., 2026); and establish reimbursement and global access pathways (Warmbrunn et al., 2024; Sharma et al., 2025). Key milestones include assay standardization, analytical and clinical validation, safety and efficacy endpoints, regulatory approval, and post-marketing surveillance (Warmbrunn et al., 2024; Chen et al., 2026; Chandra et al., 2026).

### 7.6 Cost-effectiveness considerations for microbiome-based diagnostics and therapies

Together, as mentioned above, these translational considerations complement the mechanistic and technological perspectives (Warmbrunn et al., 2024; Chen et al., 2026; Chandra et al., 2026). Cost-effectiveness is a critical determinant of clinical adoption (Sethi et al., 2023; Warmbrunn et al., 2024; Chandra et al., 2026). For diagnostics, costs include sequencing or LC–MS platforms, reagents and labor, bioinformatics, clinical validation, and regulatory submission (Chang et al., 2021; Konop et al., 2021; Kim et al., 2022; Warmbrunn et al., 2024). Potential savings arise from improved risk stratification, avoidance of unnecessary tests, and targeted therapy (Sethi et al., 2023; Kim et al., 2022; Liao et al., 2023; Warmbrunn et al., 2024; Jin et al., 2025). Economic evaluation should use cost-effectiveness and cost-utility analyses, reporting incremental cost-effectiveness ratios (ICERs) per quality-adjusted life-year (QALY), alongside budget-impact analysis (Warmbrunn et al., 2024; Chandra et al., 2026). For therapies, costs differ substantially among dietary/prebiotic interventions, probiotics, engineered live biotherapeutics, fecal microbiota transplantation, and small-molecule inhibitors of microbial enzymes (Chen et al., 2026; Zeng et al., 2026; Chandra et al., 2026). Engineered bacteria and personalized multi-omics approaches currently have high upfront costs, but automation, scale-up, and multiplexing may reduce per-patient expenses (Zeng et al., 2026; Chandra et al., 2026). Reimbursement will require prospective evidence that microbiome-guided strategies improve outcomes or reduce downstream costs (Warmbrunn et al., 2024; Chandra et al., 2026). Short-term priorities include standardized costing and early modeling (Warmbrunn et al., 2024; Chandra et al., 2026); medium-term priorities include prospective economic endpoints nested within RCTs (Iorra et al., 2026; Warmbrunn et al., 2024; Wang et al., 2024); long-term priorities include real-world evidence, value-based pricing, and reimbursement integration (Warmbrunn et al., 2024; Chandra et al., 2026).

### 7.7 Intellectual property and commercial development

Intellectual property (IP) and commercialization shape the translation of gut–heart axis technologies (Warmbrunn et al., 2024; Chandra et al., 2026). Key IP domains include microbial biomarkers and diagnostic algorithms (Kim et al., 2022; Warmbrunn et al., 2024), bacterial strains and genetic circuits (Fan et al., 2023; Mylavarapu et al., 2026; Chen et al., 2026), engineered delivery vehicles (Yu et al., 2026; Chandra et al., 2026), metabolite assays (Konop et al., 2021; Valenbreder et al., 2023), and AI-based prediction models (Chen et al., 2026; Chandra et al., 2026; Moshref-Javadi et al., 2026; Tanaka and Vécsei, 2025). Patentability challenges include the distinction between natural products and human-made modifications, diagnostic method eligibility, and sufficient disclosure of biological materials (Shi et al., 2026; Liu et al., 2024). Patent thickets, licensing complexity, and strain ownership can hinder innovation (Warmbrunn et al., 2024; Chandra et al., 2026). Commercial development requires clear regulatory pathways for live biotherapeutic products (LBPs), engineered bacteria, and combination products (Fan et al., 2023; Mylavarapu et al., 2026; Chen et al., 2026; Chandra et al., 2026); robust manufacturing under good manufacturing practice (GMP) (Mylavarapu et al., 2026; Chen et al., 2026; Chandra et al., 2026); strain stability and biocontainment (Fan et al., 2023; Mylavarapu et al., 2026; Chen et al., 2026); and scalable formulation (Yu et al., 2026; Chandra et al., 2026). Data privacy, informed consent, and benefit-sharing are also important for microbiome biobanks (Warmbrunn et al., 2024; Chandra et al., 2026). Strategies to accelerate translation include public–private partnerships, standardized licensing frameworks, open innovation for precompetitive standards, and early engagement with regulators and payers (Warmbrunn et al., 2024; Chandra et al., 2026). Balancing IP protection with data sharing will be essential to avoid monopolies and promote equitable access (Warmbrunn et al., 2024; Chandra et al., 2026).

## 8 Conclusion

The gut–heart axis is reshaping cardiovascular disease research by linking gut-derived metabolites, TMAO, short-chain fatty acids (SCFAs), and bile acids, to systemic cardiovascular regulation. These metabolites act context-dependently: TMAO promotes atherogenesis and adverse events, whereas SCFAs and certain bile acids confer anti-inflammatory and metabolic protection. Multi-omics integration across metagenomics, metabolomics, transcriptomics, and proteomics enables systems-level dissection of host–microbiome interactions, but requires standardized assays, rigorous causal inference, and validation across diverse populations to ensure reproducibility. Engineered bacterial delivery systems offer a precision therapeutic strategy for modulating specific gut metabolic pathways. Preclinical studies support their feasibility, but clinical translation is constrained by safety, stable colonization, controllability, and evolving regulatory frameworks. Future priorities include large-scale, multicenter cohort studies to validate multi-omics-derived biomarkers for risk stratification and therapeutic response, alongside synthetic biology refinements such as feedback controls and fail-safe switches. Ultimately, the gut–heart axis represents a transformative frontier in which interdisciplinary collaboration and methodological rigor can deliver precision medicine tools to improve cardiovascular outcomes worldwide.
